# Self-Supervised Contrastive Learning and GAN-Based Denoising for High-Fidelity HumanNeRF Images

**DOI:** 10.3390/s26010249

**Published:** 2025-12-31

**Authors:** Qian Xu, Wenxuan Xu, Meng Huang, Weiqing Yan, Yang Guo

**Affiliations:** 1 School of Computer and Control Engineering, Yan Tai University, Yantai 264005, China; xuwbsa@163.com (W.X.); m17538388718@163.com (M.H.); wqyan@tju.edu.cn (W.Y.); 2 School of Mechanical Engineering, Qingdao University of Technology, Huangdao District, Qingdao 266520, China; guoyang@qut.edu.cn

**Keywords:** HumanNeRF, image denoising, generative adversarial networks, contrastive learning, self-supervised learning

## Abstract

**Highlights:**

**What are the main findings?**

- This paper proposes CD-GAN, a novel self-supervised denoising framework that effectively combines contrastive learning with Generative Adversarial Networks (GANs) to remove complex, structured noise from images generated by HumanNeRF.
- The method operates without the need for any paired “clean” ground truth data by leveraging the intrinsic stochasticity of the HumanNeRF rendering process to construct positive and negative sample pairs for training.

**What are the implications of the main findings?**

- The proposed method significantly enhances the visual quality of dynamic human neural renderings by not only suppressing noise but also preserving and enhancing critical high-frequency details such as skin texture and clothing wrinkles, providing crucial support for downstream applications like virtual reality and digital avatars.
- The innovative integration of contrastive learning within a self-supervised denoising paradigm offers a new and extensible solution for addressing image quality issues in other neural rendering scenarios.

**Abstract:**

To address the prevalent noise issue in images generated by HumanNeRF, this paper proposes an image denoising method that combines self-supervised contrastive learning and Generative Adversarial Networks (GANs). While HumanNeRF excels in realistic 3D human reconstruction tasks, its generated images often suffer from noise and detail loss due to incomplete training data and sampling noise during the rendering process. To solve this problem, our method first utilizes a self-supervised contrastive learning strategy to construct positive and negative sample pairs, enabling the network to effectively distinguish between noise and human detail features without external labels. Secondly, it introduces a Generative Adversarial Network, where the adversarial training between the generator and discriminator further enhances the detail representation and overall realism of the images. Experimental results demonstrate that the proposed method can effectively remove noise from HumanNeRF images while significantly improving detail fidelity, ultimately yielding higher-quality human images and providing crucial support for subsequent 3D human reconstruction and realistic rendering.

## 1. Introduction

Since its introduction, Neural Radiance Field (NeRF) [1] has become a revolutionary technology in the field of computer graphics. NeRF employs a deep learning-based approach, mapping a scene’s color and density information into a 3D volume to generate high-quality novel view synthesis images. Unlike traditional 3D reconstruction methods, NeRF uses a neural network to represent the entire scene’s radiance transport properties, converting this 3D information into photorealistic 2D images through volumetric rendering. The revolutionary aspect of this method lies in its ability to implicitly learn a continuous 3D representation of a scene from only multi-view 2D images and synthesize new views with a high degree of realism and fine detail.

Despite NeRF’s unprecedented success in static scene view synthesis, two inherent limitations hinder its application in broader domains. First, its high computational costs for training and rendering limit its real-time effectiveness. Second, its core assumption—a static scene—makes it inadequate for dynamic worlds, especially for complex human motion, where generated images are often plagued by artifacts, motion blur, and severe detail loss. Consequently, extending NeRF to accommodate dynamic humans, complex scenes, and real-world complexities has become a major research direction.

Although HumanNeRF has made significant progress, the microscopic quality of its rendered images remains a bottleneck that needs to be addressed. In practical applications, due to the sparsity of input views, variations in lighting, and the inherent random sampling in the rendering process, the generated images are often contaminated by high-frequency noise and floating artifacts. This noise is not simple random noise but structured noise closely related to the 3D geometry and motion model. Unlike typical denoisers which assume spatially independent noise, the complexity and spatial correlation of HumanNeRF artifacts prevent traditional algorithms from achieving satisfactory results. Therefore, developing a specialized denoising algorithm that can effectively remove such complex noise while enhancing true details has become a crucial step toward the practical application of HumanNeRF.

To this end, HumanNeRF [2,3,4,5,6] emerged as a specialized technology for human 3D reconstruction and rendering based on the NeRF framework. Unlike traditional NeRF, HumanNeRF focuses on the 3D modeling and dynamic rendering of the human body, with extensive applications in virtual reality, augmented reality, film production, and game development. HumanNeRF combines NeRF’s implicit representation with a human prior model (such as Skinned Multi-Person Linear model (SMPL) to transform the unconstrained scene modeling problem into a decoupled task: learning human details in a canonical space and modeling dynamic changes in a pose space. This human-centric design allows it to more accurately capture complex human details like skin texture, clothing wrinkles, and limb movements, making the generated images more immersive and realistic from different viewpoints.

Although HumanNeRF has made significant progress in modeling macroscopic movements, the microscopic quality of its rendered images remains a bottleneck that needs to be addressed. In practical applications, due to the sparsity of input views, variations in lighting, and the inherent random sampling in the rendering process, the generated images are often contaminated by high-frequency noise and floating artifacts. This is not simple random noise but structured noise closely related to the 3D geometry and motion model. It not only severely degrades key details like skin texture and clothing wrinkles but also has a critical impact on the accuracy of downstream tasks such as pose estimation and action recognition. Therefore, developing a specialized denoising algorithm that can effectively remove such complex noise while enhancing true details has become a crucial step toward the practical application of HumanNeRF. In contrast to conventional image denoising tasks where noise is often assumed to be independent (e.g., Gaussian or Poisson noise), the noise generated during HumanNeRF rendering is far more complex: it is structured noise or floating artifacts that are strongly correlated with the 3D scene geometry and imperfect motion models. This inherent coupling makes traditional CNN-based denoisers and generic self-supervised methods prone to misinterpreting noise as high-frequency texture.

To tackle this challenge, researchers have explored various image denoising techniques. However, traditional CNN-based denoisers [7,8,9] often struggle to handle the complex structured noise in HumanNeRF. Meanwhile, supervised methods, which rely on paired “clean-noisy” data, are impractical because “clean” ground truth images are impossible to obtain. This has shifted research towards more flexible paradigms like Generative Adversarial Networks (GAN) and self-supervised learning, which offer new possibilities for learning noise distributions and recovering image details without ground truth supervision.

This paper addresses the image noise problem that arises from rendering dynamic humans with the HumanNeRF model, caused by sampling stochasticity and model uncertainty. This paper proposes a novel denoising method named CD-GAN (Contrastive Denoising GAN). Without the need for “clean” ground truth images, this method significantly improves the quality and realism of the generated images through an end-to-end framework [10,11,12] that combines self-supervised contrastive learning and generative adversarial networks.

The core architecture of CD-GAN consists of a U-Net-based generator and a PatchGAN discriminator. Its innovation lies primarily in a well-crafted joint loss function that synergistically optimizes four complementary objectives. To enable the network to learn to distinguish between “content” and “noise,” This paper designed a self-supervised contrastive loss module. This module leverages the inherent stochasticity of the HumanNeRF rendering process, using two different noisy renderings of the same pose as a positive pair and renderings of different poses as negative pairs. Through the NT-Xent contrastive loss function, this paper compel the generator’s encoder to learn a feature representation that is robust to rendering noise but highly sensitive to core content such as human pose and texture.

To enhance the realism and detail of the image while removing noise, this paper introduce an adversarial loss. The PatchGAN discriminator performs authenticity judgments on local regions (patches) of the image, effectively forcing the generator to recover high-frequency details masked by noise and avoiding the over-smoothing issue that can occur with traditional denoising methods. Furthermore, the framework integrates a content loss, comprising L1 pixel-level loss and a VGG19-based perceptual loss. The former ensures the fidelity of the generated image to the original input in its fundamental structure, while the latter ensures the consistency of image texture and style at a high-level semantic level, further improving visual quality.

This paper conducted experimental validation on the ZJU-MoCap dataset. By comparing CD-GAN with four other methods—FMGFI [13], MDBMF [14], CAFFM [15], CNNT [16], and Noise2Noise [17]—our method demonstrated superior performance. The experimental results show that our method not only effectively removes the complex noise generated by HumanNeRF rendering but also maximally preserves and enhances key details such as human skin texture and clothing wrinkles [18]. The generated images achieved significant improvements in objective metrics like *PSNR*, LPIPS [19], and *MSE*, as well as in subjective visual quality.

The innovations of this paper are as follows:

I. Proposing an innovative self-supervised denoising architecture that combines contrastive learning and generative adversarial networks.
II. Designing an efficient self-supervised contrastive learning strategy tailored to the characteristics of neural rendering.
III. Constructing a multi-objective joint optimization loss function that achieves a balance between denoising and detail enhancement.

## 2. Related Work

### 2.1. Dynamic Scenes and Human NeRF

Since the pioneering work on NeRF by Milder et al., extending it from static scenes to the dynamic world has been an active area of research in computer graphics and vision. Early explorations, such as D-NeRF [20,21,22,23], first achieved time-varying modeling of non-rigid objects by introducing a deformation field from the observation space to a canonical space. This method attributes the dynamic changes in each frame to a unified, static canonical representation, thereby capturing the object’s motion. However, for scenes with complex topological changes and large-scale movements, particularly for the human body, this single deformation field is often difficult to model precisely, easily resulting in unnatural stretching and blurring artifacts in the rendered results. Subsequent works like Nerfies [24] and HyperNeRF [25,26] improved the ability to model complex dynamic scenes to some extent by introducing more sophisticated deformation models, such as elastic deformation and hypernetwork-based topology-aware deformations, but at the cost of significantly increased model complexity and training time.

**Figure 1:**
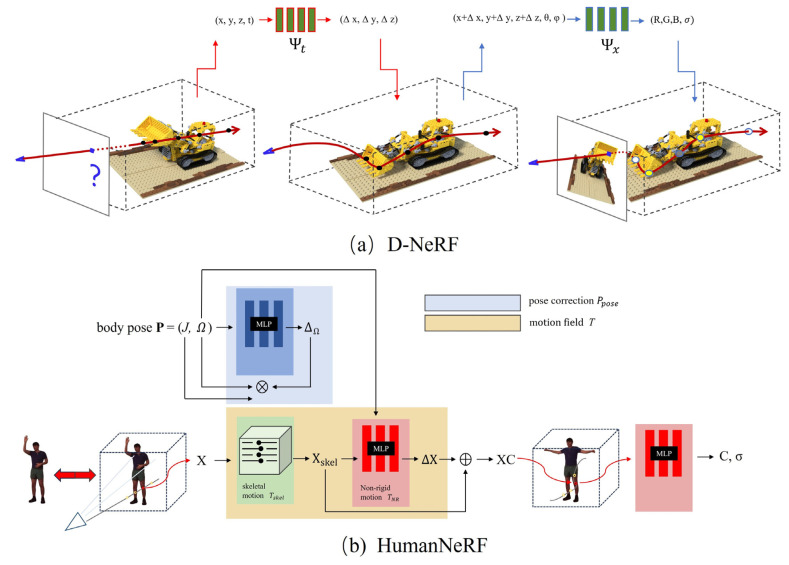
(**a**) D-NeRF: This model uses a time index t to learn a single deformation field, mapping observation space (x,y,z,t), to a canonical space. (**b**) HumanNeRF: This model decouples motion by utilizing pose parameters (P) and motion fields ($T_{skel\;}$ for skeletal motion, $T_{NR}$ for non-rigid motion) to deform points X into a canonical space XC, where the final MLP is queried to output color and density (C,σ). Note: The symbol ⊕ denotes the summation of deformation vectors (ΔX).

Considering the highly structured nature of human motion and the availability of prior knowledge, researchers quickly shifted to modeling methods specifically targeting the human body in pursuit of higher-fidelity results. The core idea of these methods is to deeply integrate NeRF’s implicit neural representation with parametric human models, such as SMPL [27,28]. Pioneering works like Animatable NeRF [29] and HumanNeRF decoupled the complex dynamic human modeling problem into two more manageable sub-problems: first, learning pose-independent static human appearance (e.g., body shape, clothing details) in a canonical space under a standard pose; second, accurately driving the motion and deformation of points in the canonical space using pose parameters provided by the SMPL. This decoupling strategy greatly simplifies the learning task, allowing the model to focus on capturing high-frequency surface details. Building on this, works like Neural Body [30] and Neural Actor [31] further explored how to better fuse multi-view information, handle complex clothing dynamics, and generate freely editable human avatars. Figure 1 illustrates the methodological comparison in motion modeling between general-purpose dynamic NeRF and human-specific NeRF.

Despite the remarkable success of these specialized HumanNeRF models in capturing macroscopic motion and overall structure, the microscopic quality of their rendered images remains a challenge. Particularly in cases of sparse input views, poor lighting conditions, or rapid motion, the generated images are often plagued by high-frequency noise and floating artifacts due to model uncertainty and random sampling during the rendering process. This severely impacts the representation of key details such as skin texture and clothing wrinkles. Effectively resolving this quality bottleneck is the core problem that this research aims to address.

### 2.2. Deep Learning-Based Image Denoising

Image denoising is a fundamental and enduring research topic in the field of computer vision. In recent years, deep learning-based methods have become the dominant paradigm in this field, by virtue of their powerful feature learning capabilities. These methods can be broadly categorized into two major classes: supervised and self-supervised/unsupervised. To better illustrate the classification of existing image denoising methods, Table 1 summarizes the mainstream denoising paradigms and their primary limitations when applied to HumanNeRF scenarios, thereby highlighting the unique positioning of the method proposed in this paper.

Recent advancements continue to refine deep learning-based methods across diverse image processing tasks. For instance [32], explored novel approaches leveraging deep networks for high-fidelity image reconstruction, further emphasizing the shift towards data-driven restoration paradigms.

**Table 1:** Categorization of Related Work on Image Denoising Methods.

Category	Representative Works	Core Idea	Key Limitation
Supervised Denoising	DnCNN [33], FFDNet [34]	End-to-end mapping learned from paired “clean-noisy” data	Relies on clean ground truth, which is unavailable in NeRF scenes
General Self-supervised Denoising	Noise2Noise [35], Noise2Self [36]	Training using the statistical properties of noise	Struggles with structured noise related to geometry
**Specialized Self-supervised Denoising**	**CD-GAN**	**Combines contrastive learning and GANs, trained using rendering stochasticity**	

#### 2.2.1. CNN-Based Supervised Denoising

Early deep learning-based denoising methods primarily relied on supervised learning. Representative works such as DnCNN [33] and FFDNet [34] achieved significant success in handling synthetic noise like Gaussian white noise by designing deep Convolutional Neural Networks (CNNs) trained end-to-end on a large number of paired “clean-noisy” images. These networks learn a residual mapping from the noisy image to its corresponding clean image. The U-Net architecture, with its encoder–decoder structure and skip connections, excels at preserving image details and has been widely used in image restoration tasks. However, the core bottleneck of these supervised methods is their strong dependence on paired training data. In neural rendering scenarios like HumanNeRF, This paper cannot obtain perfectly “clean” ground-truth images as supervision signals because the true scene geometry and lighting are unknown. Therefore, these supervised methods cannot be directly applied to our problem.

#### 2.2.2. Self-Supervised and Unsupervised Denoising

To overcome the dependency on clean images, researchers have proposed a series of self-supervised and unsupervised denoising methods. Among these, the seminal work Noise2Noise [35] theoretically demonstrated that training with only paired “noisy–noisy” images (i.e., two independent noise realizations of the same scene) can achieve performance comparable to training with “clean-noisy” pairs. Building on this, Noise2Self [36] and blind-spot-based strategies advanced this concept further, requiring only a single noisy image. By cleverly designing the network architecture or training strategy to prevent the network from accessing a target pixel’s value when predicting it, they enable self-supervised training without any paired data.

These self-supervised methods have provided significant inspiration for our work, particularly the “noisy–noisy” pair concept from Noise2Noise, which aligns with our strategy of constructing positive pairs using rendering stochasticity. However, a core assumption of these general-purpose self-supervised methods is that the noise is spatially conditionally independent between pixels and has a zero mean (e.g., Gaussian or Poisson noise). In contrast, the noise generated during HumanNeRF rendering is far more complex: it is often structured noise or floating artifacts that are strongly correlated with the 3D scene geometry, surface materials, and imperfect motion models. This noise is not spatially independent. Consequently, directly applying general-purpose self-supervised denoising methods may struggle to fully model this complex noise distribution, leading to incomplete denoising or the introduction of new artifacts. This indicates the need for a specialized self-supervised learning framework tailored to the noise characteristics of HumanNeRF.

While frameworks like SCONE-GAN [10] leverage contrastive learning for general image translation by focusing on semantic consistency, they are not designed to handle noise tied to 3D geometric variance. Our key distinction lies in the task-specific design of the self-supervised signal. We uniquely exploit the inherent stochasticity of volumetric rendering to construct positive pairs (same content, different noise realizations) and use pose changes to define negative pairs. This forces the encoder to learn a representation that is robust to 3D sampling noise yet sensitive to pose and identity, a capability essential for denoising neural human renderings that general contrastive GANs lack.

### 2.3. Quality Enhancement and Denoising for NeRF

Although Neural Radiance Fields have achieved great success in view synthesis, their rendering quality, particularly for dynamic and complex scenes, remains suboptimal. To address this issue, current research primarily follows two technical paths: one is to improve the NeRF model and rendering process itself, and the other is to perform post-processing enhancement on the rendered results from NeRF.

Some works are dedicated to enhancing NeRF’s representation capability and rendering quality at the source. For example, mip-NeRF [37] effectively alleviates the aliasing problem caused by scale changes by modeling conical frustums instead of sampling single points along rays, thus generating sharper images. Ref-NeRF [38], on the other hand, achieves realistic rendering of high-gloss effects such as specular reflections through fine-grained modeling of the view direction. Additionally, some works introduce regularization terms to smooth NeRF’s geometry or appearance fields to reduce floating artifacts. While these methods improve rendering quality to some extent, they usually come at the cost of increased model complexity and training time, and they cannot completely eliminate all the noise and artifacts caused by insufficient input data (such as sparse views) or the model’s inherent uncertainty.

## 3. Methodology

### 3.1. The Overall Framework of CD-GAN

**Figure 2:**
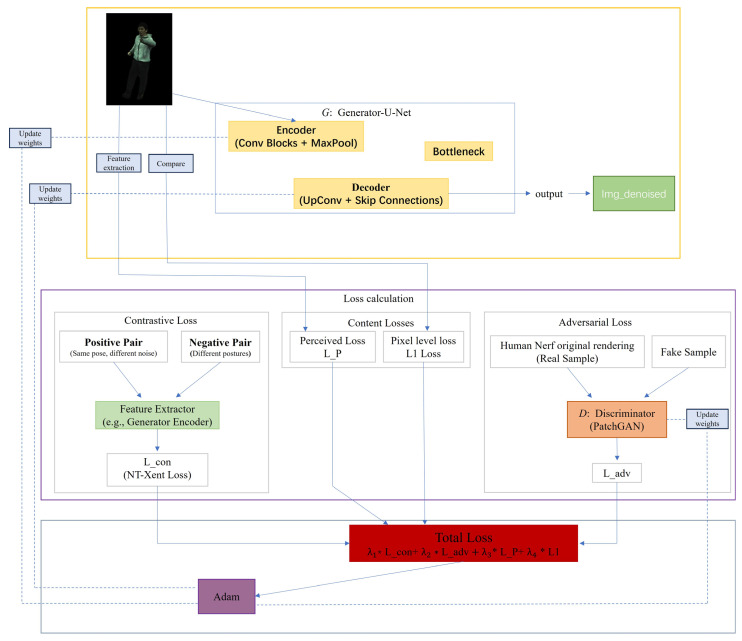
Overview of the proposed CD-GAN framework. Our method consists of a U-Net-based generator (yellow part) and a PatchGAN discriminator (orange part). The entire framework is guided by a joint loss function for self-supervised training, which includes contrastive loss to disentangle content from noise; content loss (pixel-level $L_{1}$ loss and perceptual loss *L_P*) to ensure content fidelity; and adversarial loss to enhance the realism and details of the generated image. All modules are collaboratively optimized, without requiring any clean ground-truth images.

This sections details the self-supervised denoising framework that we proposed to address the HumanNeRF rendering noise problem—CD-GAN. The core objective of this framework is to map an image $I_{noisy}$, which is rendered by the HumanNeRF model and contains complex structured noise, to a high-quality, detail-rich denoised image $I_{denoised}$ via a deep generative network. The design of the entire framework follows a self-supervised learning paradigm, meaning that no paired “clean-noisy” ground truth data is required during the training process. The overall framework of our proposed CD-GAN is illustrated in Figure 2. The framework primarily consists of two core modules: a generator *G*, based on the U-Net architecture, which performs the non-linear mapping from the noisy image to the denoised image. It is responsible for understanding image content, identifying and removing noise, while also reconstructing details masked by the noise. A discriminator *D*, based on the PatchGAN architecture, whose role is to evaluate the local realism of the generated image $I_{denoised}$. By comparing it with the “real” noisy images, it provides an adversarial gradient to the generator to enhance the realism and high-frequency details of the generated result. To effectively train these two networks, this paper have designed a well-crafted joint loss function. This function guides the entire framework through a multi-objective, collaborative optimization process. It combines adversarial training to enhance realism, self-supervised contrastive learning to decouple content from noise, and content preservation constraints (including pixel-level and perceptual-level) to ensure image fidelity. The following sections will provide a detailed introduction to the specific network architectures and each of the loss functions.

### 3.2. Detailed Explanation of Network Architecture

Our proposed CD-GAN framework consists of a generator network *G* and a discriminator network *D*. This section will detail their respective network architectures and design considerations.

#### 3.2.1. Generator Network

To effectively achieve the mapping from a noisy image to a clean one while preserving rich spatial details, our generator *G* adopts the classic U-Net architecture [39]. U-Net, with its symmetric encoder–decoder structure and hallmark skip connections, excels in image-to-image translation tasks. Its primary components include:

**I.**  Encoder: The encoder part is responsible for extracting multi-scale, hierarchical features from the input image. It consists of a series of repeated convolutional blocks, each containing two 3 × 3 convolutional layers, followed by a Rectified Linear Unit (ReLU) activation function and batch normalization [40]. After each convolutional block, this paper uses a 2 × 2 max-pooling layer for downsampling, which halves the spatial resolution of the feature maps while doubling the number of feature channels. This process allows the network to progressively expand its receptive field, transitioning from capturing low-level edge and texture information to understanding higher-level semantic content.
**II.**  Decoder: The goal of the decoder part is to progressively restore the abstract features extracted by the encoder into a high-resolution image. Its structure is symmetric to the encoder and is implemented through a series of upsampling blocks. Each upsampling block first uses a 2 × 2 transposed convolution to double the resolution of the feature map, then, through skip connections, concatenates the feature map of the current decoder layer with the feature map from the corresponding level of the encoder along the channel dimension. This step is crucial as it directly “injects” the high-resolution, low-level details captured early by the encoder into the decoding process, greatly mitigating the information loss caused by downsampling, and is essential for reconstructing sharp edges and fine textures. The concatenated feature map is then processed through two 3 × 3 convolutional layers.
**III.**  Output Layer: In the final layer of the decoder, this paper use a 1 × 1 convolutional layer to map the multi-channel feature map back to a three-channel RGB image. Finally, a Tanh activation function is used to normalize the output pixel values to the range of [−1, 1].

#### 3.2.2. Discriminator Network

To guide the generator to produce visually more realistic and detail-rich images, this paper employ a discriminator network *D* based on PatchGAN [40]. Unlike traditional discriminators that output a single “real/fake” probability for the entire image, PatchGAN operates in a fully convolutional manner, outputting an N × N feature map for the input image. Each element in this feature map corresponds to the authenticity judgment of a specific-sized local region of the input image. The primary advantage of selecting PatchGAN is that it forces the generator to produce details that conform to the real data distribution across all local regions of the image, not just in terms of global structural similarity. This powerful constraint on local textures and high-frequency details effectively prevents the generator from producing blurry or overly smooth images, which is highly beneficial for improving the clarity and realism of the denoised results. In our specific implementation, the discriminator is composed of a series of convolutional layers, with each layer followed by a LeakyReLU activation function. Unlike conventional classification networks, this paper do not use batch normalization in the intermediate layers of the discriminator to avoid introducing unnecessary sample-wise dependencies. The final convolutional layer outputs a single-channel feature map, which is used for calculating the adversarial loss.

### 3.3. Joint Loss Function

The training of the CD-GAN framework is governed by a joint loss function composed of four parts, which is pivotal for mapping noisy images to high-quality, clear results. This function facilitates the synergistic optimization of complementary objectives, guaranteeing that the final output strikes an optimal trade-off between realism, content fidelity, and detail clarity. Consequently, the total loss function, $L_{total}$, is expressed as a weighted summation of these individual terms:

(1) $$L_{total}=\lambda_{1}\;\times \;L\_\mathrm{con}+\lambda_{2}\;\times \;L\_\mathrm{adv}+\lambda_{3}\;\times \;L\_P+\lambda_{4}\;\times \;L_{1}$$

Here, *λ*_1_, *λ*_2_, *λ*_3_, and *λ*_4_ are hyperparameters used to balance the importance of each loss term. Next, this paper will provide a detailed introduction to each loss term.

#### 3.3.1. Adversarial Loss

The adversarial loss is the foundation of GAN training. It enhances the realism of the generated images through a minimax game between the generator *G* and the discriminator *D*. This paper adopts the standard GAN loss function. For the discriminator *D* [41], its objective is to maximize its ability to distinguish between real images and generated images. Its loss function is defined as:

(2) $$L_{D}=E_{x\sim \;p_{data\;}(x)}\left[\log\;D(x)]+E_{z\sim p_{z}(z)}[\log(1\;-\;D(G(z)))\right]$$

Here, $E_{x\sim \;p_{data\;}(x)}$ represents a sample from the real data distribution (i.e., the original noisy rendered image from HumanNeRF which acts as the ‘real’ target for the discriminator), and $E_{z\sim p_{z}(z)}$ represents the noisy input image fed into the generator *G* from the input data distribution. Its loss function is defined as:

(3) $$L_{adv}=E_{z\sim p_{z}(z)}[log(1-D(G(z)))]$$

By minimizing $L_{adv}$, the generator is forced to learn the data distribution of real images, thereby producing Images that are more realistic in texture and lighting details.

#### 3.3.2. Self-Supervised Contrastive Loss

This is the core innovation of our method [42], aimed at enabling the network to autonomously learn to distinguish between the “content” and “noise” of an image in the absence of clean ground truth.

Sample Construction: The core innovation of our contrastive module is its tailoring to the neural rendering pipeline. The method leverages the inherent sampling stochasticity of the HumanNeRF rendering process. Specifically, for any fixed human pose and camera viewpoint, we generate two independent noisy renderings ($I_{pos\_a},I_{pos\_b}$). This strategy guarantees that the pair shares identical underlying 3D content while featuring uncorrelated noise realizations, forming our positive pair. This design is crucial as it specifically guides the network to discard the stochastic rendering component while retaining the deterministic content features (pose, identity). Concurrently, images corresponding to different poses are randomly sampled from the training set to serve as negative examples.

Loss Calculation: The sample pairs are first processed by a feature extractor, whose weights are shared with the generator’s encoder, $G_{enc}$, to produce latent representations. Subsequently, the contrastive loss is computed using the NT-Xent (Normalized Temperature-scaled Cross-Entropy Loss) function [43]. Specifically, for a given positive pair (*i*,*j*) within a mini-batch, the loss is formulated as:

(4) $$L_{i,j}=-\log\frac{exp(sim(z_{i},z_{j})/\tau )}{\sum_{k=1}^{2N}1_{\left[k\neq i\right]}exp(sim(z_{i},z_{k})/\tau )}$$

Here, *z =* $G_{enc}$ represents the extracted feature vector, *sim*() is the cosine similarity, τ is the temperature hyperparameter, and $1_{\left[k\neq i\right]}$ is an indicator function used to exclude the sample itself. The total contrastive loss, *L_con*, is the average of the losses for all positive pairs in a mini-batch. Through this loss, the network is driven to learn a feature representation that is robust to rendering noise but sensitive to core content such as human pose and identity, which is fundamental to achieving high-quality denoising.

We experimentally set the temperature hyperparameter τ to 0.07. This value is widely recognized in contrastive learning literature [42] for enforcing fine-grained feature separation. Given that the HumanNeRF structured noise is highly correlated with true content, this small τ is necessary to ensure the network imposes a strong metric constraint, thereby effectively separating the subtle noise features from the core content features (human pose and identity) during optimization.

#### 3.3.3. Content Preservation Loss

To ensure that the generator does not distort the original image content while denoising, this paper introduce two types of content preservation losses.

**I.**  Pixel-level Loss: This paper uses the $L_{1}$ loss [44] to constrain the similarity between the generated image and the input image at the pixel level. *L*_1_ loss tends to produce sharper edges, and its definition is:

(5) $$L_{1}=E_{I_{noisy}}\sim D_{noisy}[||G\left(I_{noisy}\right)-I_{noisy}{\left|\right|}_{1}]$$

This loss ensures the fidelity of the generated image to the input in terms of low-level structure and color.

**II.**  Perceptual Loss: To preserve consistency in high-level semantics and textural style, a perceptual loss [45] is employed. This is achieved using a VGG19 network [46,47], pre-trained on ImageNet with its weights frozen, which is denoted as Φ. The perceptual loss is then defined as the Euclidean distance between the feature maps of the generated and input images, extracted from specific intermediate layers of the VGG network:

(6) $$L\_P=E_{I_{noisy}}\sim D_{noisy}[||\Phi_{j}\left(G\left(I_{noisy}\right)\right)-\Phi_{j}\left(I_{noisy}\right){\left|\right|}_{2}^{2}]$$

Here, $\Phi_{j}$ represents the feature map of the j-th layer of the VGG19 network. This loss effectively prevents the image from becoming blurry and better preserves high-level textures such as skin texture and clothing wrinkles, $E_{I_{noisy}}\sim D_{noisy}$ denotes the expectation taken over the empirical distribution $D_{noisy}$ of all noisy input images $I_{noisy}$ used for training.

### 3.4. Methodological Discussion

This section discusses the design philosophy of our proposed CD-GAN framework at a deeper level, including its unique “understand-regenerate” mechanism under the self-supervised paradigm, as well as the synergy and balance among the multi-objective loss functions.

#### 3.4.1. Noise Disentanglement and Content Generation Under the Self-Supervised Paradigm

The proposed CD-GAN framework’s core advantage lies in its ability to cleverly combine the two tasks of Noise Disentanglement and Content Generation within a completely self-supervised paradigm. Traditional denoising methods often treat denoising as a signal separation problem. In contrast, our method, through contrastive loss, compels the generator’s encoder to learn a feature representation that is insensitive to noise. This feature can be regarded as a “clean,” content-rich latent representation. Subsequently, the generator’s decoder, under the joint guidance of adversarial and perceptual loss, acts as a high-quality content generator. It takes this “clean” latent feature and renders it into a detail-rich, texturally realistic image. Therefore, our framework does not simply “remove” noise but performs a more intelligent two-stage process of “understand-purify-regenerate,” which gives it an advantage over traditional methods when dealing with structured noise that is tightly coupled with content.

#### 3.4.2. Synergy and Balance of Multi-Objective Losses

**Figure 3:**
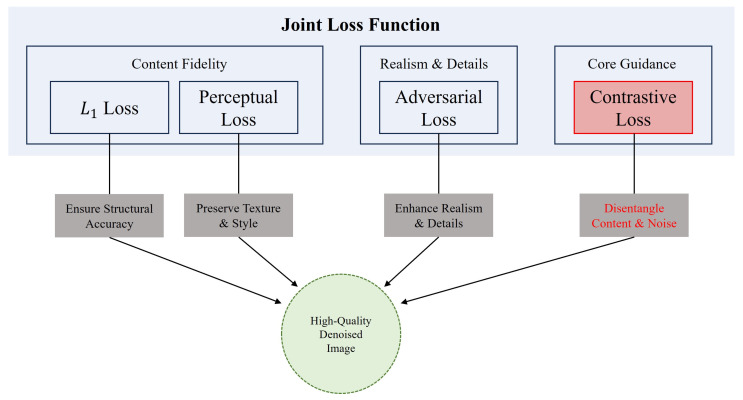
The synergistic mechanism of the joint loss function in CD-GAN. Our joint loss function is composed of four functionally complementary parts that collectively guide the model to generate high-quality denoised images. The Content Fidelity module, through L1 loss and perceptual loss, ensures the accuracy of the generated image in its low-level structure and high-level texture. The Realism & Details module utilizes adversarial loss to enhance the image’s realism and high-frequency details. Most critically, the contrastive loss (highlighted in red) in the Core Guidance module is responsible for fundamentally decoupling content from noise. The synergy of these four losses ensures a comprehensive improvement in the final output’s structure, texture, realism, and content purity.

Another key to our method’s success is the multi-objective joint loss function this paper designed. These four losses are not a simple functional superposition but form a functionally complementary, mutually balancing organic whole, as illustrated in Figure 3. The $L_{1}$ loss and perceptual loss together form the cornerstone of content preservation. They ensure the fidelity of the generated result to the original input from both low-level pixel structure and high-level semantic texture, forming the foundation for stable model training. On this basis, the adversarial loss is responsible for enhancing the final realism, driving the model to create subtle high-frequency details that conform to real physical laws but might be ignored by the $L_{1}$ and perceptual loss, serving as the finishing touch to improve the image’s visual quality. Meanwhile, the contrastive loss, as the core of our method, plays the crucial role of a guiding rudder. By fundamentally improving the model’s ability to discriminate noise, it ensures that the entire learning process always moves in the right direction of distinguishing content from noise, and it creates the prerequisite for other loss functions to work more effectively. This ingenious synergistic mechanism allows our model to flexibly find an ideal balance point among the seemingly contradictory goals of denoising, fidelity, and detail enhancement by adjusting the loss weights, thereby achieving a comprehensive high-quality output.

## 4. Experiments

### 4.1. Datasets and Training Details

#### 4.1.1. Datasets Preprocessing

Our experiments are based on the public ZJU-MoCap dataset [28]. For this paper, we selected 6 subjects with diverse motions (313, 377, 386, 387, 390, 392, 393, 394). These subjects were specifically chosen to cover a wide range of clothing topologies (e.g., tight-fitting sportswear vs. loose hoodies) and motion complexities, ensuring a representative and challenging evaluation of the model’s robustness across different scenarios. This dataset provides high-resolution dynamic human sequences captured by 23 synchronized cameras from multiple viewpoints, offering high-quality data for training and evaluating dynamic human 3D reconstruction models. The original ZJU-MoCap dataset includes the raw image sequence for each camera view (e.g., Camera_B1 to Camera_B23), as well as the corresponding camera parameters, SMPL parameters (lbs, params), and 2D keypoint information (keypoints2d). To make this data suitable for training our CD-GAN framework, we first performed a series of preprocessing steps to convert it into a more standardized format. Our preprocessing mainly includes the following steps:

**I.**  **Data Filtering and Reorganization:** We extracted the image sequences and corresponding camera intrinsic and extrinsic parameters for each camera view from the original dataset. For ease of management, we stored all views’ images uniformly in an “images” folder.
**II.**  **Foreground Mask Generation:** To enable the model to focus on the human subject and ignore background interference, we generated precise foreground masks for each image frame. These binary masks accurately segment the human silhouette and are uniformly stored in a masks folder. During training, these masks are used to ensure that ray sampling and loss calculation are performed only within the human body region.
**III.**  **Parameter Integration and Packaging:** We integrated the camera parameters, SMPL pose parameters, and pre-calculated human mesh information for all frames and packaged them into .pkl and .yaml files, such as cameras.pkl and mesh_infos.pkl. This packaging process not only improves data loading efficiency but also makes the entire dataset structure clearer and more modular.

**Figure 4:**
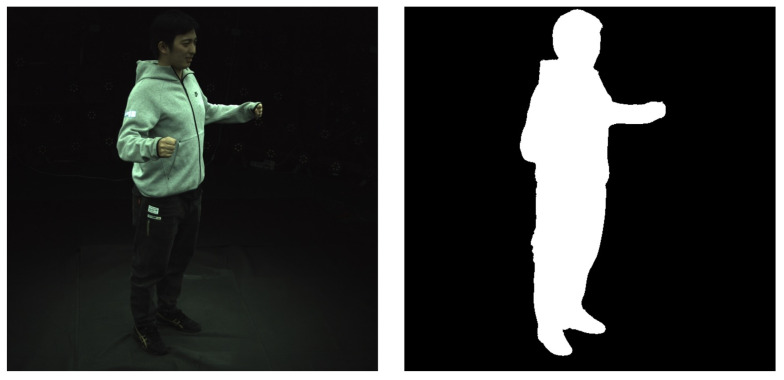
Example of a single-frame image after preprocessing. (**Left**) The RGB image in the “images” folder. (**Right**) The corresponding binary foreground mask in the “masks” folder.

Through these preprocessing steps, we constructed a standardized, easy-to-use dataset for the subsequent training of the HumanNeRF model and the CD-GAN denoising framework. Figure 4 shows an example of a single-frame image from the “images” folder and its corresponding Mask image from the “masks” folder after preprocessing.

#### 4.1.2. Training Details

We employed a consistent split strategy for the selected ZJU-MoCap subjects. For each subject’s image sequence, we reserved the first 80% of the frames for the training set, the next 10% for the validation set, and the last 10% for the testing set. This temporal splitting method ensures that the model is evaluated on future poses and motions not seen during training, providing a fair assessment of generalization capability.

To implement the CD-GAN framework proposed in this paper, we use PyTorch as the deep learning framework and conduct all experiments on a single NVIDIA RTX 5070Ti (12G) GPU. The entire training process was performed on our preprocessed dataset, iterating for a total of 400 K steps to ensure both the generator and discriminator could fully learn and converge. For the training strategy, we employed the Adam optimizer [48] to optimize the network parameters. For the core Multi-Layer Perceptron (MLP) in the generator, which is responsible for learning the canonical space color and density, we set a higher initial learning rate of 5 × 10^−4^. In contrast, for the non-rigid deformation MLP, pose decoder, and motion weight decoder, we set a smaller learning rate of 0.00005 to achieve more stable fine-tuning. To optimize the training dynamics, we used an exponential decay learning rate strategy, with the decay rate set to 500. We maintained the stability of the adversarial training through several key design choices. The use of PatchGAN [40], which inherently focuses on local realism, significantly stabilizes the training process compared to full-image discriminators. Furthermore, the overall joint loss function, particularly the synergy between the adversarial term $L_{adv}$ and the highly constrained content preservation terms ($L_{1}\;and\;L_{P}$), acts as an effective regularizer that prevents mode collapse. In addition, we employed an exponential decay learning rate strategy (as detailed above) to ensure smooth and balanced convergence of both the generator and discriminator.

To balance memory usage and training efficiency, we adopted a patch-based ray sampling strategy, where each training batch processes 1 sample, and each sample contains 6 image patches of size 20 × 20. For volumetric rendering along each ray, we used a hierarchical sampling strategy, setting 64 coarse sampling points and 128 fine sampling points (N_samples). Additionally, to enhance the model’s robustness, we introduced ray perturbation during the training process.

In terms of the loss function, we experimentally set the weight for the Mean Squared Error (*MSE*) in the content loss to 0.2 and the weight for the perceptual loss (LPIPS) to 1.0 (lossweights). During the testing phase, all renderings were performed on a black background (bgcolor: [0.0, 0.0, 0.0]) to ensure consistency in evaluation. This combination of parameters not only optimized the training process but also ensured the high quality of the rendered output.

### 4.2. Evaluation Metrics

According to standard settings, this paper uses Peak Signal-to-Noise Ratio (*PSNR*) to measure image quality and Mean Squared Error (*MSE*) to evaluate the difference between predicted and actual values. Their calculation formulas are as follows:

(7) $$\mathrm{PSNR}=10\times \log_{10}\left(\frac{MAX_{I}^{2}}{MSE}\right)=20\times \log_{10}(\frac{MAX_{I}}{\sqrt{MSE}})$$

where $MAX_{I}$ represents the maximum possible pixel value of the image.

(8) $$\mathrm{MSE}=\frac{1}{N}\sum_{i=1}^{N}{\left(Y_{i}-{\hat{Y}}_{i}\right)}^{2}$$

where *N* is the total number of pixels in the image, $Y_{i}$ is the ground truth value of the *i*-th pixel, and $Y_{i}$ is the predicted value of the *i*-th pixel.

Furthermore, to evaluate image similarity in a way that better aligns with human visual perception, this paper also employs the Learned Perceptual Image Patch Similarity (LPIPS) as a key evaluation metric. Unlike *MSE*, LPIPS measures the perceptual difference between two images by calculating the distance between them in the feature space of a deep neural network. The calculation of LPIPS is not based on a simple mathematical formula but is instead realized through a deep neural network.

### 4.3. Ablation Study

To systematically validate the contribution of each component within the CD-GAN framework, we conducted a comprehensive ablation study on Subject 387. The quantitative results are presented in Table 2. Initially, the raw HumanNeRF renderings exhibit a relatively low *PSNR* of 27.21 dB. The baseline model, trained exclusively with pixel-level *L*_1_ loss, achieves a moderate improvement to 28.53 dB; however, it suffers from a high LPIPS score of 0.0641, indicating an over-smoothed appearance insufficient for fine detail recovery. By incorporating the adversarial loss (*L__adv_*) in the ‘w/o Contrastive’ variant, the *PSNR* improves to 29.62 dB, yet it still significantly lags behind the full model, highlighting the limitation of standard GAN training in handling structured noise.

Most critically, the comparison between the “w/o Contrastive” variant and our full model reveals that omitting the contrastive loss (*L__con_*) results in a substantial *PSNR* drop of 2.6 dB (from 32.22 dB to 29.62 dB). This empirically verifies that our self-supervised strategy is indispensable for robustly disentangling noise from content. Furthermore, removing the adversarial loss (“w/o Adversarial”) leads to a degradation in perceptual quality (LPIPS increases from 0.0502 to 0.0534), confirming the discriminator’s role in synthesizing realistic high-frequency features. Consequently, the complete CD-GAN framework achieves superior performance across all metrics (*PSNR*: 32.22 dB, *MSE*: 0.0006, LPIPS: 0.0502), successfully striking an optimal balance between effective denoising and high-fidelity detail preservation.

### 4.4. Quantitative Analysis

We conducted a comprehensive quantitative evaluation on the ZJU-MoCap dataset comparing our method with four existing denoising approaches (FMGFI [13], MDBMF [14], CAFFM [15], CNNT [16], Noise2Noise [34]) and the classic self-supervised baseline Noise2Noise. To ensure a fair comparison and improve readability, Table 3 summarizes the average performance across all six test subjects. For detailed quantitative results of each individual subject, please refer to Table in the Appendix A. From the average results in Table 3, we can clearly observe that the proposed CD-GAN method consistently outperforms all comparison methods across all evaluation metrics. Specifically, on the *PSNR* metric, CD-GAN achieved the highest score of 30.46 dB, surpassing the second-best method (MDBMF) by approximately 1.25 dB. On the *MSE* metric, our method achieved the lowest error (0.0009), indicating superior accuracy in signal restoration. Notably, in terms of perceptual quality measured by LPIPS, CD-GAN still demonstrates a clear advantage with the lowest score of 0.0508. This confirms that by incorporating contrastive learning with adversarial training, our method is capable of better preserving high-level semantic features and generating images that are closest to the ground truth in terms of human visual perception.

### 4.5. Qualitative Analysis

**Figure 5:**
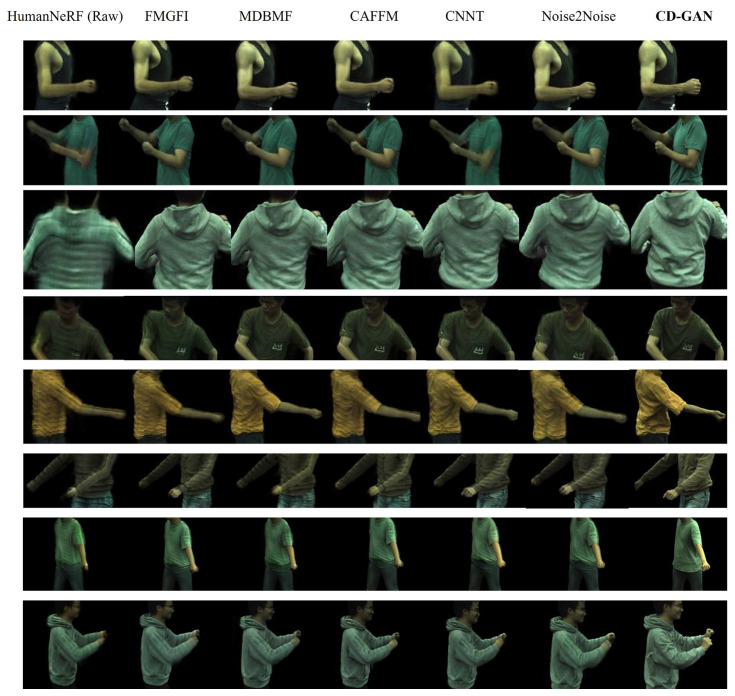
Qualitative comparison results on 6 diverse moving subjects (313, 377, 386, 387, 390, 392, 393, 394) from the ZJU-MoCap dataset.

To more visually evaluate and compare the denoising performance of different methods, in Figure 5, we present the qualitative comparison results of CD-GAN against four comparison methods on multiple sequences from the ZJU-MoCap dataset. In the figure, each row represents a different test sequence, and each column displays the result of one method. It can be clearly observed from the figure that all comparison methods (FMGFI, MDBMF, CAFFM, CNNT) face varying degrees of challenges in removing noise. In contrast, the proposed CD-GAN method achieves the best visual results in all showcased cases. Our model not only thoroughly removes the complex noise generated by HumanNeRF rendering but, more importantly, also successfully preserves and enhances crucial high-frequency details. For instance, as shown in the third row, CD-GAN clearly restores the wrinkles and shading transitions on the back of the sweatshirt; in the fifth row, it accurately reconstructs the contour of the arm and the texture of the T-shirt; and in the final row, its restoration of the color and wrinkles of the jeans is also the most authentic. These qualitative results robustly demonstrate the superiority of our method. By combining self-supervised contrastive learning with generative adversarial networks, CD-GAN learns to precisely distinguish between content and noise. This allows it to generate images with greater realism and richer detail while effectively denoising, resulting in a visual quality that is significantly superior to all comparison methods.

## 5. Limitations and Future Work

While CD-GAN achieves state-of-the-art denoising performance, we acknowledge that optimization for efficiency was not the primary focus during the initial design phase. Our main objective was to establish the feasibility of achieving high-fidelity detail restoration under the complex self-supervised paradigm. Consequently, the use of a robust U-Net architecture for the generator introduces a non-trivial computational overhead during inference. This current cost places the total processing time outside the requirements for real-time applications (30 FPS), a limitation viewed as a necessary trade-off for maximizing output quality.

Beyond the real-time bottleneck, our method is subject to several additional limitations:

**I.**  Computational Overhead: As a post-processing step based on a U-Net architecture, CD-GAN adds significant computational cost during inference compared to direct rendering, limiting its applicability in real-time HumanNeRF applications.
**II.**  Generalization to Extreme Cases: Our current model may underperform in highly complex scenarios not well represented in the ZJU-MoCap dataset, such as scenes with extremely sparse input views, rapidly changing dynamic lighting, or highly transparent clothing materials.
**III.**  Domain Specificity: The contrastive learning strategy is tightly coupled with the stochasticity of neural rendering, requiring specific data preparation (e.g., generating noisy–noisy pairs), which may hinder immediate transferability to other general image-to-image tasks.

## 6. Conclusions

This paper addresses the common problems of image noise and detail loss in rendering dynamic humans with HumanNeRF by proposing a novel self-supervised image denoising framework named CD-GAN. The core of this method lies in the ingenious combination of self-supervised contrastive learning and generative adversarial networks, thereby achieving high-quality enhancement of rendered results without the need for any “clean” ground-truth images. We first leverage the intrinsic randomness of the NeRF rendering process to design an efficient contrastive learning strategy, which allows the model to autonomously learn to decouple the essential content of an image from complex structured noise. Building on this, we construct a multi-objective joint loss function composed of adversarial loss, content preservation loss, and contrastive loss. Through collaborative optimization, this function not only effectively removes noise but also, driven by adversarial training, significantly enhances and restores high-frequency details masked by noise. Extensive qualitative and quantitative experiments conducted on the public ZJU-MoCap dataset have fully validated the effectiveness and superiority of our method. The experimental results demonstrate that, compared to several existing denoising methods, CD-GAN achieves the best performance across all metrics, including *PSNR*, *MSE*, and LPIPS, and generates images that are visually clearer and more realistic. Therefore, future work will focus on the following directions: Firstly, in terms of efficiency and real-time performance: We can explore lightweighting the denoising framework or integrating it more tightly with the NeRF rendering process to reduce the additional computational overhead introduced by post-processing. This would allow it to meet the demands of real-time rendering applications while maintaining high-quality output. Secondly, in terms of model generalization and application extension: We can enhance the model’s robustness against extreme lighting conditions and complex clothing materials by training on more diverse datasets. Furthermore, the ideas of this framework can be extended to a broader range of neural rendering scenarios, such as denoising for large-scale urban scenes or more complex non-rigid objects.

## Figures and Tables

**Table 2:** Ablation study on the contribution of different loss components using Subject 387 from the ZJU-MoCap dataset. The symbol “√” indicates that the corresponding loss component is included. Bold indicates the best performance, while “↑” and “↓” denote that higher or lower values are better, respectively.

Model Variant	L1	L_adv	L_con	L_P	*PSNR* ↑	*MSE* ↓	LPIPS ↓
HumanNeRF (Raw)	—	—	—	—	27.21	0.0019	0.0892
Baseline (L1 only)	√	—	—	—	28.53	0.0014	0.0641
w/o Contrastive	√	√	—	√	29.62	0.0011	0.0640
w/o Adversarial	√	—	√	√	30.46	0.0009	0.0534
w/o Perceptual	√	√	√	—	30.97	0.0008	0.0515
CD-GAN (Ours)	√	√	√	√	**32.22**	**0.0006**	**0.0502**

**Table 3:** Quantitative comparison on ZJU-MoCap dataset (where higher *PSNR* values are better, lower *MSE* values are better, and bold indicates the best results).

Method	*PSNR* ↑	*MSE* ↓	LPIPS ↓
FMGFI	28.56	0.0015	0.0731
MDBMF	29.10	0.0013	0.0694
CAFFM	28.05	0.0015	0.0681
CNNT	27.74	0.0017	0.0806
Noise2Noise	29.07	0.0013	0.0697
**CD-GAN**	**30.89**	**0.0008**	**0.0415**

## Data Availability

The data that support the findings of this study are openly available in ZJU-MoCap at: [https://zju3dv.github.io/zju_mocap/] (accessed on 22 December 2025).

## Appendix A. Detailed Quantitative Results

		Subject 377			Subject 386	
	*PSNR* ↑	*MSE* ↓	LPIPS ↓	*PSNR* ↑	*MSE* ↓	LPIPS ↓
FMGFI	28.54	0.0014	0.062	29.24	0.0012	0.0765
MDBMF	28.86	0.0013	0.0763	31.55	0.0007	0.0303
CAFFM	28.24	0.0015	0.0705	30.97	0.0008	0.0323
CNNT	25.68	0.0027	0.0752	27.96	0.0016	0.0877
Noise2Noise	30.00	0.0010	0.0404	28.54	0.0014	0.0625
**CD-GAN**	**30.97**	**0.0008**	**0.0036**	**33.98**	**0.0004**	**0.0285**
		Subject 387			Subject 392	
	*PSNR* ↑	*MSE* ↓	LPIPS ↓	*PSNR* ↑	*MSE* ↓	LPIPS ↓
FMGFI	29.21	0.0012	0.0784	27.45	0.0018	0.0737
MDBMF	30.00	0.0010	0.0553	29.59	0.0011	0.0705
CAFFM	31.55	0.0007	0.0777	28.24	0.0015	0.0671
CNNT	30.46	0.0009	0.0596	28.54	0.0014	0.0847
Noise2Noise	31.55	0.0007	0.0622	28.86	0.0013	0.0539
**CD-GAN**	**32.22**	**0.0006**	**0.0502**	**30.00**	**0.0010**	**0.0233**
		Subject 393			Subject 394	
	*PSNR* ↑	*MSE* ↓	LPIPS ↓	*PSNR* ↑	*MSE* ↓	LPIPS ↓
FMGFI	26.02	0.0025	0.0884	28.54	0.0014	0.0676
MDBMF	28.24	0.0015	0.0831	26.99	0.0020	0.1052
CAFFM	26.77	0.0021	0.0848	28.24	0.0015	0.0886
CNNT	27.96	0.0016	0.1132	29.59	0.0011	0.0605
Noise2Noise	27.77	0.0017	0.1125	27.45	0.0018	0.0860
**CD-GAN**	**29.21**	**0.0012**	**0.0791**	**30.46**	**0.0009**	**0.0601**
		Subject 313			Subject 390	
	*PSNR* ↑	*MSE* ↓	LPIPS ↓	*PSNR* ↑	*MSE* ↓	LPIPS ↓
FMGFI	29.50	0.0012	0.0710	30.00	0.0010	0.0669
MDBMF	28.50	0.0015	0.0700	29.00	0.0013	0.0647
CAFFM	24.50	0.0023	0.0650	25.92	0.0019	0.0591
CNNT	25.00	0.0022	0.0850	26.69	0.0018	0.0786
Noise2Noise	28.90	0.0013	0.0720	29.52	0.0011	0.0679
**CD-GAN**	**30.10**	**0.0009**	**0.0450**	**30.14**	**0.0009**	**0.0422**
